# Melatonin Enhances the Therapeutic Effect of Plasma Exosomes Against Cerebral Ischemia-Induced Pyroptosis Through the TLR4/NF-κB Pathway

**DOI:** 10.3389/fnins.2020.00848

**Published:** 2020-08-18

**Authors:** Kankai Wang, Junnan Ru, Hengli Zhang, Jiayu Chen, Xiao Lin, Zhongxiao Lin, Min Wen, Lijie Huang, Haoqi Ni, Qichuan Zhuge, Su Yang

**Affiliations:** ^1^Zhejiang Provincial Key Laboratory of Aging and Neurological Disorder Research, The First Affiliated Hospital of Wenzhou Medical University, Wenzhou, China; ^2^Department of Neurosurgery, The First Affiliated Hospital of Wenzhou Medical University, Wenzhou, China

**Keywords:** ischemic stroke, melatonin, plasma exosome, pyroptosis, inflammatory response, TLR4/NF-κB signaling pathway

## Abstract

**Introduction:**

Ischemic stroke-induced inflammation and inflammasome-dependent pyroptotic neural death cause serious neurological injury. Nano-sized plasma exosomes have exhibited therapeutic potential against ischemia and reperfusion injury by ameliorating inflammation. To enhance its therapeutic potential in patients with ischemic injury, we isolated exosomes from melatonin-treated rat plasma and assessed the neurological protective effect in a rat model of focal cerebral ischemia.

**Methods:**

Basal plasma exosomes and melatonin-treated plasma exosomes were isolated and intravenously injected into a rat model of focal cerebral ischemia. Neurological recovery was evaluated by determining the modified neurological severity score (mNSS), infarct volume, and brain water content. Pyroptosis in the ischemic cortex was detected through dUTP nick-end labeling (TUNEL) assay, lactate dehydrogenase (LDH) release, and gasdermin D (GSDMD) cleavage. NLRP3 inflammasome assembly and global inflammatory cytokine secretion were detected by enzyme-linked immunosorbent assay (ELISA) and Western blot assay. In immunized Sprague–Dawley rats, microglia pyroptosis was determined through a positive percentage of IBA1^+^ and caspase-1 (p20)^+^ cells. Finally, the microRNA (miRNA) profiles in melatonin-treated plasma exosomes were analyzed by exosome miRNA microarray analysis.

**Results:**

Melatonin treatment enhanced plasma exosome therapeutic effects against ischemia-induced inflammatory responses and inflammasome-mediated pyroptosis. In addition, we confirmed that ischemic stroke-induced pyroptotic cell death occurred in the microglia and neuron, while the administration of melatonin-treated exosomes further effectively decreased the infarct volume and improved recovery of function *via* regulation of the TLR4/NF-κB signaling pathway. Finally, the altered miRNA profiles in the melatonin-treated plasma exosomes demonstrated the regulatory mechanisms involved in neurological recovery after ischemic injury.

**Conclusion:**

This study suggests that nano-sized plasma exosomes with melatonin pretreatment might be a more effective strategy for patients with ischemic brain injury. Further exploration of key molecules in the plasma exosome may provide increased therapeutic value for cerebral ischemic injury.

## Introduction

According to the Global Burden of Disease Study, stroke has become the second leading cause of death and the third leading cause of global disability-adjusted life years, putting tremendous pressure on medical care worldwide (Feigin et al., 2014). As a sudden neurological dysfunction caused by interruption of the blood supply, various pharmacological and cytological therapies have been applied to mitigate post-stroke injury and improve recovery of neurological function. Growing evidence suggests that the inflammatory response and inflammatory cell death play crucial roles in the secondary injury in ischemic stroke (Jin et al., 2010). Although a vast number of systemic studies have been devoted to elucidating the internal mechanisms, there is still missing information and the mechanism content is constantly updated.

Pyroptosis was identified as a programmed form of necrosis, which features a lytic process featuring cell swelling and large bubbles emanating from the plasma membrane (Sarhan et al., 2018). As a high level of inflammatory response, pyroptosis is triggered by nucleotide-binding oligomerization domain-like receptors (NLRs), which recognize various pathological stimuli, including pathogen-associated molecular patterns (PAMPs) and endogenous damage-associated molecular patterns (DAMPs). Important compounds of the inflammasome are the NLRPs (NOD-like receptors containing pyrin domains), which consist mainly of NLRP3 and NLRP1. NLRP3 interacts with pro-caspase-1 upon connection with the apoptosis-associated speck-like protein containing the CARD (ASC) adapter, leading to the cleavage of precursors of inflammatory cytokines into mature forms, including interleukin-18 (IL-18) and interleukin-1β (IL-1β) (He et al., 2016). Activated caspase-1 subsequently cleaves and releases the N-domain of gasdermin D (GSDMD), which eventually forms membrane pores and releases mature inflammatory mediators into the extracellular space, causing a severe inflammatory cascade reaction (Liang and Liu, 2016). Studies have shown that inflammation-related pyroptosis is an important factor affecting stroke prognosis (Jin et al., 2010). In a rat stroke model, the protein GSDMD promptly increased and then climbed to a peak 24–48 h post-ischemia, indicating that pyroptosis occurs efficiently during cerebral ischemia injury (Zhang D. et al., 2019).

Exosomes are nano-sized extracellular vesicles (EVs) secreted by living cells into the extracellular fluids, and biofluids such as blood, urine, and cerebrospinal fluid have all been proven to secrete exosomes (Zhang et al., 2015). Recently, exosomes have received widespread attention for their inclusive composition (Zhang X. et al., 2019). These nanocarriers contain proteins, lipids, and nucleic acids, which are the major mediators of cell–cell communication and play regulatory roles in several cellular processes such as immune regulation (Vanni et al., 2017; Zhang X. et al., 2019). In addition, plasma exosomes from young individuals have been reported to have anti-inflammatory effects on ischemia (Lee B.R. et al., 2018); nevertheless, the mechanism by which exosomes protect nerve cells from ischemia injury remains unclear. Numerous studies have confirmed that the microRNAs (miRNAs) in exosomes function as key factors in the regulatory process of inflammation, angiogenesis, cellular transport, apoptosis, and proteolysis (Eirin et al., 2014; Liu et al., 2016). A class of exosomal miRNAs such as microRNA-29a, microRNA-9, microRNA-30c-5p, microRNA-30d, and microRNA-155 have been proven to modulate the cell pyroptotic process after brain injury (Li et al., 2014; Jeyabal et al., 2016; Wu et al., 2016; Minghua et al., 2018; Ding et al., 2020). Previous studies have shown altered miRNA profiles in the plasma or exosomes in various physiological environments or after pharmacological treatments (Minghua et al., 2018; Yoon et al., 2020; Zhang and Jin, 2020). Therefore, it is of significant interest to determine the composition and function of exosomal miRNAs in inflammasome-dependent pyroptosis during cerebral ischemic injury.

Melatonin is a potent free radical scavenger and broad-spectrum antioxidant that is synthesized by the pineal gland or other organs and has been shown to inhibit inflammation and apoptosis in cerebral ischemic injury (Rancan et al., 2018). However, the specific mechanism has not yet been clearly explained. Interestingly, some studies have found that melatonin can affect the composition of exosomes, such as microRNA (Cheng et al., 2017; Alzahrani, 2019; Hunsaker et al., 2019; Yoon et al., 2020), suggesting that it has versatile therapeutic means to improve stroke prognosis. In this study, we investigated the therapeutic effect of plasma exosomes against ischemia-induced inflammation and inflammasome-dependent pyroptosis. Based on these findings, we suggest that melatonin alters the microRNA profiles in plasma exosomes and ultimately improves stroke recovery. However, the correlation between the anti-pyroptotic mechanisms and the specific miRNA functions requires further research.

## Materials and Methods

### Animals and Ethics Statement

Sprague–Dawley rats weighing 200–250 g were purchased from the Shanghai Charles River Experimental Animal Limited Liability Company (Shanghai, China). The animals were housed in a standardized animal care center with appropriate temperature and humidity with a 12 h light/dark cycle. Rats were given free access to food and water. All experimental procedures were approved by the Ethics Committee of Wenzhou Medical University and were carried out in strict accordance with the animal care and use guidelines of the National Institutes of Health.

### Stroke Model and Exosome Injection

**FIGURE 1:**
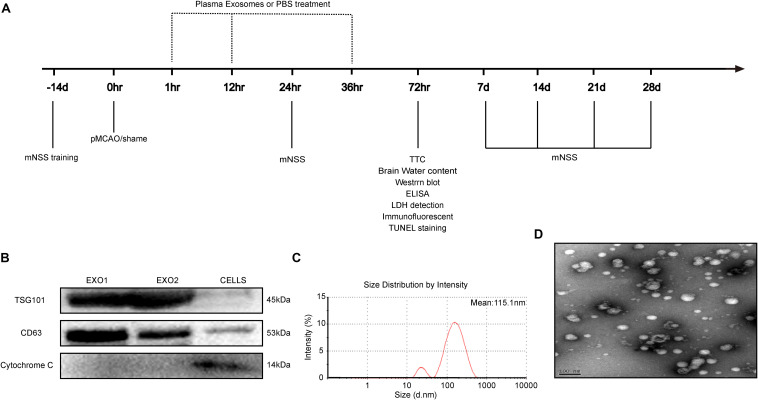
Time axis and characterization of exosomes. **(A)** Experimental design time axis of this study. **(B)** Western blot analysis of the specific exosome, including TSG101, CD63, and cytochrome C. **(C)** Particle size distribution measured by nanoparticle tracking analysis system. **(D)** Morphology observed by transmission electron microscope.

The permanent distal middle cerebral artery occlusion (pMCAO) model was used to create permanent focal ischemia, as previously described (Ren et al., 2018). Briefly, Sprague–Dawley rats were anesthetized and incised between their left eye socket and tragus. With the temporal muscles separated, a 3 mm diameter bone window was drilled for left middle cerebral artery exposure. After separation and blocking of the common carotid arteries on both sides, the distal middle cerebral artery was cauterized for permanent blocking. The carotid arteries were then released, and wounds were sutured with continual vital signs monitoring. The sham group underwent the same surgical procedure without cerebral artery blocking. To explore the influence of exosomes (Exo) and melatonin-Exo on pMCAO-induced cerebral injury, exosomes derived from rat plasma suspended in phosphate-buffered saline (PBS) were injected intravenously through the tail vein at a concentration of 100 μg per rat at 1, 12, and 36 h after cerebral artery blocking. The pMCAO group received an equal volume of PBS. All experiments followed the schedule shown in Figure 1A.

### Isolation and Characterization of Serum Exosomes

Melatonin dissolved in 0.9% saline (with 5% DMSO) was injected intraperitoneally once a day for seven consecutive days (10 mg/kg). At day 8, the rats were sacrificed and blood was collected for plasma acquisition. Plasma exosomes were then extracted with a Total Exosome Isolation Kit (Invitrogen). The isolated exosomes were suspended in PBS for further characterization. Exosome size distribution and concentration were analyzed using a ZETASIZER Nano series-Nano-ZS system. Vesicles were visualized by light scattering using a Hitachi H-7650 transmission electron microscope. Specific exosome markers including TSG101, CD63, and cytochrome C were determined through Western blot analysis.

### TTC Staining

Three days after stroke, the brain was excised and cut into five pieces. The sections were then immersed in preheated 2% 2,3,5-triphenyltetrazolium chloride (TTC; Sigma Aldrich) solution for 20 min. After washing with PBS, the sections were placed with the frontal pole toward the front and captured with a camera. Finally, the infarct volume was calculated by ImageJ software.

### Neurobehavioral Training and Evaluation

The modified neurological severity score (mNSS) test was used to assess neurological deficits, as described previously (Longa et al., 1989). The mNSS includes multiple tasks to assess motor, sensory, reflex, and balance abilities and is conducted at 1, 7, 14, 21, and 28 days after surgery, with a maximum deficit score of 18. A higher score indicates more severe neurological dysfunction. All mice were pre-trained for 14 days before the operation until their performance reached a steady state.

### Assessment of Cerebral Edema

Brain water content (BWC) was evaluated as previously described (Ishrat et al., 2015). Rat brain tissues were taken 3 days after surgery, and the ipsilateral and contralateral sides were then separated and weighed for wet weight (WW). The tissues were dried in an oven at 100°C for 24 h to obtain a dry weight (DW). BWC was calculated using the following formula: 100% × (WW − DW)/WW.

### Western Blot Analysis

Total proteins were extracted from brain tissues using RIPA lysis buffer (Thermo Fisher Scientific, United States) and quantified with a bicinchoninic acid (BCA) Protein Assay Kit (Thermo Fisher Scientific, United States) according to the manufacturer’s instructions. Sodium dodecyl sulfate polyacrylamide gel electrophoresis (SDS-PAGE) was then conducted. After being transferred onto polyvinylidene fluoride (PVDF) membranes, the membranes were blocked with 5% milk for 2 h, followed by incubation at 4°C for 24 h with primary antibodies against the following proteins: α-tubulin (1:1,000, CST, Shanghai, China); NLRP3 (1:1,000, CST, Shanghai, China); ASC (1:1,000, Santa Cruz, Shanghai, China); caspase-1 (1:1,000, CST, Shanghai, China); GSDMD (1:1,000, Abcam, Cambridge, United Kingdom); TLR4 (1:1,000, Abcam, Cambridge, United Kingdom); and NF-κB (1:1,000, Abcam, Cambridge, United Kingdom). The membrane was further incubated with a secondary antibody and then detected using the Bio-Rad ChemiDoc XRS imaging system. ImageJ software was used to analyze immunoreactive bands. The target protein signal intensities were compared to either the α-tubulin or β-actin intensity.

### Enzyme-Linked Immunosorbent Assay and Serum LDH Detection

The secretion levels of TGF-β, TNF-α, IL-6, IL-1β, IL-10, IL-18, TLR4, HMGB1, and NF-κB were measured using an enzyme-linked immunosorbent assay (ELISA) kit (Beyotime, Shanghai, China) according to the manufacturer’s instructions. Plasma lactate dehydrogenase (LDH) detection was measured using an LDH activity detection kit (Solarbio, Beijing, China).

### Immunofluorescence

Brain sections were fixed with 4% paraformaldehyde at room temperature for 20 min and then incubated in PBST (0.4% triton in PBS) containing 5% bovine serum albumin (BSA) solution (Santa Cruz, Shanghai, China) for 30 min to block non-specific staining. Next, the sections were incubated with primary antibodies at 4°C overnight, followed by incubation with the corresponding secondary antibody at 37°C for 1 h. The nuclei were counterstained with DAPI (Abcam). Then, the sections were examined with a scanning fluorescence microscope (Leica Microsystems) and analyzed using ImageJ software.

### TdT-Mediated dUTP Nick-End Labeling Assays

A One Step dUTP nick-end labeling (TUNEL) Apoptosis Assay Kit (Beyotime, Shanghai, China) was used to detect cell apoptosis according to the manufacturer’s instructions. Briefly, the cells were incubated with 50 μl TUNEL detection mixture at 37°C for 1 h in the dark and rinsed in PBS. Subsequently, 50 μl streptavidin–horseradish peroxidase (HRP) solution was added and incubated for 30 min at room temperature. DAB staining was then performed, followed by hematoxylin staining of the nuclei.

### miRNA Microarray Analysis

The NEB Next Multiplex Small RNA Library Prep Set for Illumina (NEB, United States) was used for RNA library preparation according to the manufacturer’s protocol. Briefly, total RNA extracted from each plasma exosome sample was ligated with 3′ and 5′ adapters for Illumina. After sample quality control and quantification with a NanoDrop ND-1000, reverse transcription was conducted using ProtoScript II Reverse Transcriptase (NEB, United States). An Agilent 2100 Bioanalyzer was used for library quality control and quantification. The cDNA library for 135–155 bp (corresponding to single-end small RNA at 15–35 nt) was selected for miRNA sequencing with an Illumina NextSeq 500 instrument (Illumina Inc., United States). Solexa CHASTITY was used for quality screening of clean reads, and the 3′ ends of the sequenced reads were removed to obtain trimmed reads that were at least 15 nucleotides in length. Filtered reads were annotated using miRDeep2. Altered levels of the miRNA expressions (normalized) in exosomes were calculated with fold change > 1.5 and *P*-values < 0.05 (Student’s *t*-test).

### Target Prediction and Bioinformatics Analysis

The sequencing data have been deposited in the NCBI Gene Expression Omnibus (GEO^1^) with the accession number GSE147578.

To detect the top expressed miRNAs in plasma exosomes and alternative expressions after melatonin treatment, edgeR was used to identify differentially expressed genes. All predicted genes with a target prediction score ≥ 80 were subjected to Gene Ontology (GO) for gene enrichment and functional annotation analyses.

### Statistical Analysis

GraphPad Prism software was used for the statistical analyses. The measured data are expressed as the mean ± SD. Differences between the two groups were evaluated by Student’s *t*-test. Analysis of variance (ANOVA) was used to compare three or more groups. *P* < 0.05 was considered statistically significant.

## Results

### Isolation and Identification of Exosomes

Adult male rats (200–250 g) received intraperitoneal injections for seven consecutive days with a control solvent (5% DMSO + PBS) or melatonin (10 mg/kg, dissolved in 5% DMSO + PBS). On day 8, plasma exosomes (EXO1 represented basic plasma exosomes and EXO2 represented melatonin-treated plasma exosomes) were isolated and identified. Concurrently, cell extraction from the microglial BV2 cell line was selected as a control for exosome identification. We detected the characteristic biomarkers of exosomes, TSG101 and CD63, which were expressed at higher levels in the exosomes than in the control cells, while cytochrome C (negative marker for exosomes) was absent or underrepresented in the exosomes (Figure 1B). The spherical morphology characteristics and a diameter of 30–150 nm with an average particle size of 115.1 nm were confirmed through nanoparticle tracking analysis (NTA) (Figure 1C) and transmission electron microscope (TEM) (Figure 1D) and were in agreement with other studies (Lotvall et al., 2014). Taken together, these data demonstrate that the isolates had all the characteristics of purified exosomes.

### Melatonin Enhances the Therapeutic Effect of Exosomes by Reducing Brain Damage and Promoting Functional Recovery After Stroke

**FIGURE 2:**
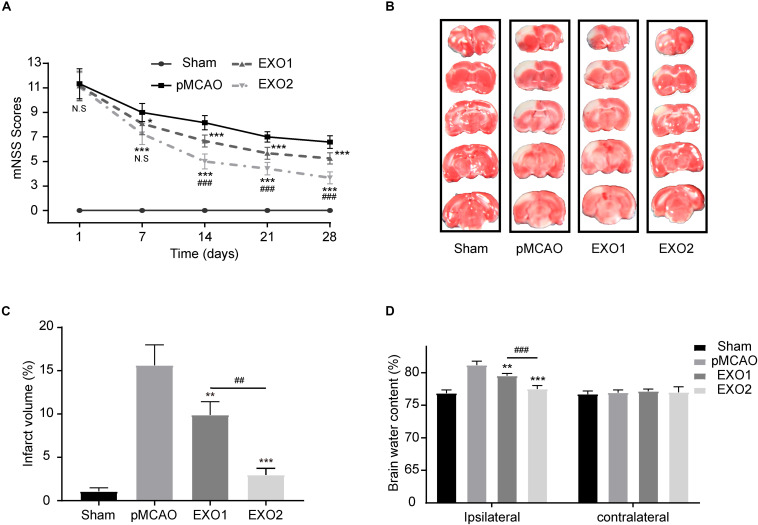
Exosome treatment reduced brain damage and promoted functional recovery after stroke. **(A)** Neurological recovery was evaluated by the modified neurological severity score (mNSS) at 1, 7, 14, 21, and 28 days post-permanent distal middle cerebral artery occlusion (pMCAO, *n* = 12). **(B,C)** Representative images of 2,3,5-triphenyltetrazolium chloride (TTC) staining in five sequential brain slices from the various groups of rats (*n* = 3). **(D)** Brain water content was evaluated at 72 h post-injury (*n* = 4). Data are presented as the mean ± SD. *P < 0.05, **P < 0.01, ***P < 0.001 *vs*. the pMCAO group. ^##^*P* < 0.01, ^###^*P* < 0.001 *vs*. the EXO1 (basic plasma exosomes) group.

Studies have confirmed the therapeutic effect of young rat plasma exosomes on cardiac ischemia–reperfusion injury and angiogenesis (Vicencio et al., 2015; Chen et al., 2020). To investigate the effect of melatonin on exosomes in the treatment of ischemic stroke, plasma exosomes from melatonin- or PBS-treated adult rats were prepared for treatment. As shown in Figure 2, the EXO1 group (pMCAO with EXO1 treatment, 100 μg) and the EXO2 group (pMCAO with EXO2 treatment, 100 μg) had lower mNSS than the pMCAO group (equal volume of PBS treatment). At 28 days after stroke, the EXO1 group score was 5.25 ± 0.4523, which was significantly lower than that of the pMCAO group, approximately 6.583 ± 0.5149 (*P* < 0.001). Whereas the score of the EXO2 group was even lower at 3.667 ± 0.4924, which was more significant than that of the EXO1 group (*P* < 0.001), indicating that the EXO2 group received better therapeutic action. Similarly, the TTC staining assay showed that the cerebral infarction of the EXO1 group exhibited a smaller infarct size of 9.915 ± 1.504% compared to 15.64 ± 2.354% in the pMCAO group (*P* < 0.05). The infarct size decreased even further to 2.98 ± 0.7406% in the EXO2 group (*P* < 0.01, compared with EXO1), suggesting that melatonin enhanced the effect of exosomes on reducing the infarct volume. Moreover, the BWC assay showed that the brain water content of the EXO1 and EXO2 groups decreased to 79.525 ± 0.358% (*P* < 0.01, compared with the pMCAO group) and 77.505 ± 0.527% (*P* < 0.001, compared with the EXO1 group), respectively, compared with the pMCAO group (81.178 ± 0.584) and sham group (76.840 ± 0.515), suggesting that EXO2 is more significant for the prognosis of stroke in rats. Therefore, we believe that melatonin enhanced the plasma exosomes’ ability to improve neural functional recovery.

### Melatonin-Exosomes Protected Against Ischemia-Induced Pyroptosis Through an NLRP3-Mediated Pathway

**FIGURE 3:**
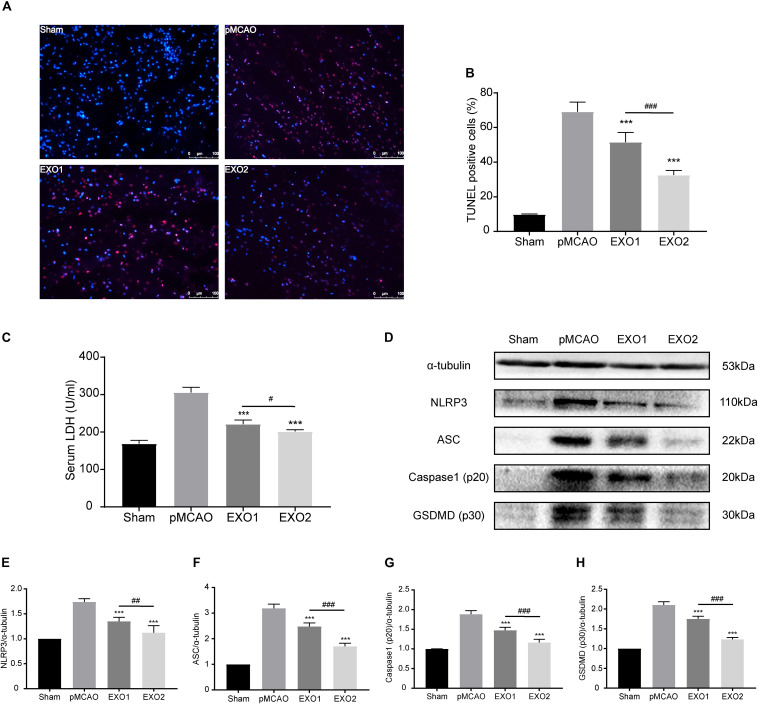
Administration of basic plasma exosomes (EXO1) and melatonin-treated plasma exosomes (EXO2) inhibited NLRP3-dependent pyroptosis in the ischemic cortex. **(A)** dUTP nick-end labeling (TUNEL) assay was used to detect the apoptosis of cortical nerve cells post-ischemic injury. Representative images of the TUNEL-positive apoptotic cells (*red*) in sagittal brain sections at day 3 post-injury. The nuclei of all cells were stained with DAPI (*blue*, *n* = 5. **(B)** Comparison of the number of TUNEL-positive cells with EXO1 or EXO2 treatment. **(C)** Lactate dehydrogenase (LDH) release for the detection of cell membrane pore formation (*n* = 5). **(D–H)** Western blot analysis of NLRP3, ASC, active caspase-1, and active GSDMD (N-terminal, *n* = 5). Data are presented as the mean ± SD. ***P < 0.001 *vs*. the permanent distal middle cerebral artery occlusion (pMCAO) group. ^#^*P* < 0.05, ^##^*P* < 0.01, ^###^*P* < 0.001 *vs*. the EXO1 group.

Inflammation-related pyroptosis is reported to play an important role in the secondary injury of stroke (Zhang D. et al., 2019). Studies have confirmed that pyroptosis reached its peak at 24–48 h post-stroke, then gradually decreased (Shi et al., 2017; Evavold et al., 2018). To investigate the regulatory roles of melatonin (MT)-exosomes in the pyroptotic process, we analyzed the anti-apoptosis effect after ischemic stroke. The TUNEL assay showed a noticeable decrease in apoptotic death in the EXO1 group, from approximately 69.11 ± 5.508% to 51.5 ± 5.679%. Meanwhile, the EXO2 group exhibited a more significant suppression of cell death (*P* < 0.01, compared with the EXO1 group) (Figures 3A,B), indicating that melatonin enhanced the inhibitory effect of adult rat plasma exosomes against ischemia-induced cell death. In addition, cleavage of GSDMD (p30) (Figures 3D,H) and increased LDH secretion (Figure 3C) after cerebral ischemia indicated GSDMD membrane pore formation (Bortolotti et al., 2018) and cellular content release. With regard to GSDMD and LDH, the EXO2 group showed a more significant reduction compared with the EXO1 group, indicating that MT-exosomes are more conducive to the prevention of pyroptotic cell death in the pMCAO model. Moreover, by detecting the NLRP3–caspase-1 axis (Figures 3D–G), the results revealed that the NLRP3 inflammasome assembled proteins, including NLRP3, ASC, and caspase-1 (p20), were all decreased after exosome administration, and the EXO2 group showed a larger inhibitory action on NLRP3 inflammasome assembly (*P* < 0.01, compared with the EXO1 group). These results demonstrated that adult plasma exosomes can inhibit NLRP3-mediated pyroptosis after stroke, and the inhibitory effect could be strengthened with melatonin treatment.

### Melatonin-Treated Exosomes Reduced Microglia and Neuron Pyroptosis in an Ischemic Stroke Model

**FIGURE 4:**
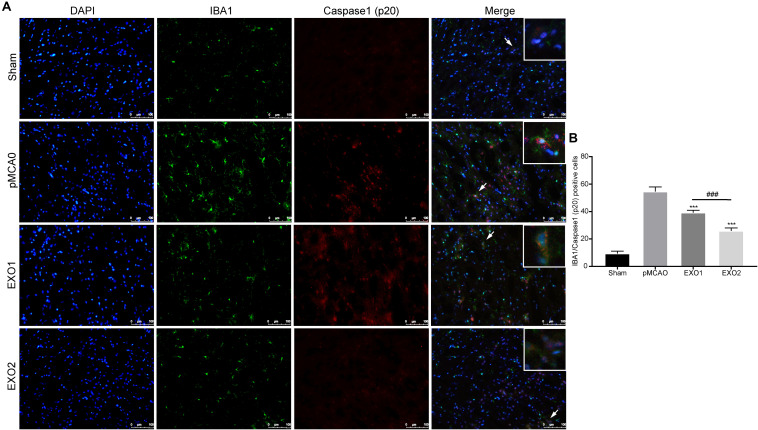
Melatonin-treated exosomes ameliorated microglia pyroptosis in permanent distal middle cerebral artery occlusion (pMCAO) rats. **(A)** Representative photographs of double immunofluorescence staining for Iba-1 (*green*) and cleaved caspase-1 (*red*) in the cortex at 72 h post-injury. **(B)** Statistical analysis of the immunofluorescence-positive cells. *Scale bars*, 100 μm (*n* = 5). Three sections were taken from each rat, and three fields were randomly selected within the penumbra area of each section. After cell counting, the average value was calculated and used for statistical analysis. Data are presented as the mean ± SD. ***P < 0.001 vs. the pMCAO group. ^###^*P* < 0.001 vs. the EXO1 (basic plasma exosomes) group.

It has been proven that inflammatory pyroptosis in the brain after stroke is mainly mediated by the microglia (Mamik and Power, 2017; Fricker et al., 2018). Therefore, we performed immunofluorescence staining of the brain tissue to explore microglial pyroptosis. The results revealed that the number of pyroptotic microglia in the pMCAO group was approximately 54.56 ± 3.39, which was significantly higher than that in the sham group and was in accordance with a previous study. After treatment with EXO1 and EXO2, the pyroptotic numbers decreased to 39.22 ± 1.698 and 25.89 ± 2.137, respectively (*P* < 0.001, IBA1 stains the microglia and caspase-1 stains pyroptotic cells) (Figure 4), which were in accordance with the former results. These results indicate that plasma exosomes pretreated with melatonin can better inhibit the occurrence of microglial pyroptosis, thereby blocking the initiation of the inflammatory response after stroke.

**FIGURE 5:**
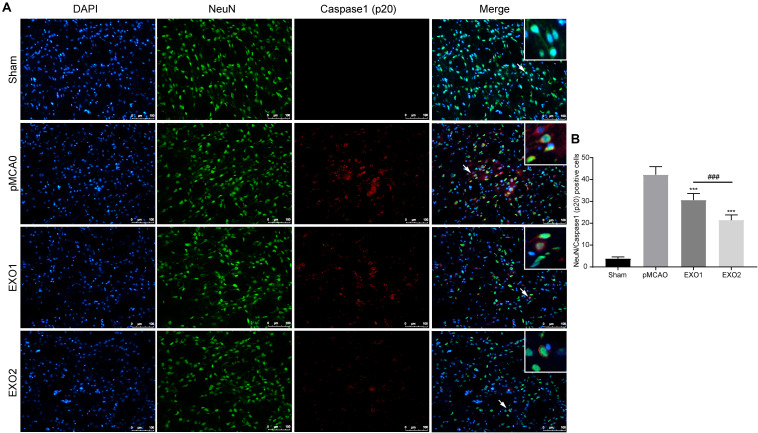
Melatonin-treated exosomes ameliorated neuron pyroptosis in permanent distal middle cerebral artery occlusion (pMCAO) rats. **(A)** Representative photographs of double immunofluorescence staining for NeuN (*green*) and cleaved caspase-1 (*red*) in the cortex at 72 h post-injury. **(B)** Statistical analysis of the immunofluorescence-positive cells. *Scale bars*, 100 μm (*n* = 5). Three sections were taken from each rat, and three fields were randomly selected around the infarction area of each section. After cell counting, the average value was calculated and used for statistical analysis. Data are presented as the mean ± SD. ***P < 0.001 *vs*. the pMCAO group. ^###^*P* < 0.001 *vs*. the EXO1 (basic plasma exosomes) group.

The anti-pyroptotic role in neurons was also detected. A former result (Figure 3) has declared the protective role of exosomes in preventing neuronal death in ischemic penumbra. Here, we detected the pyroptosis occurrence in neurons around the infarction area. The result showed that the number of pyroptotic neurons in the pMCAO group was 42.24 ± 3.65, while with treatments of EXO1 and EXO2, the pyroptotic numbers decreased to 30.64 ± 3.00 and 21.49 ± 2.36, respectively (Figure 5), indicating a more powerful protective effect of the melatonin-treated exosomes in preventing neurons from pyroptotic death.

### Melatonin-Treated Exosomes Alleviated the Post-stroke Inflammatory Response Through the TLR4/NF-κB Pathway

**FIGURE 6:**
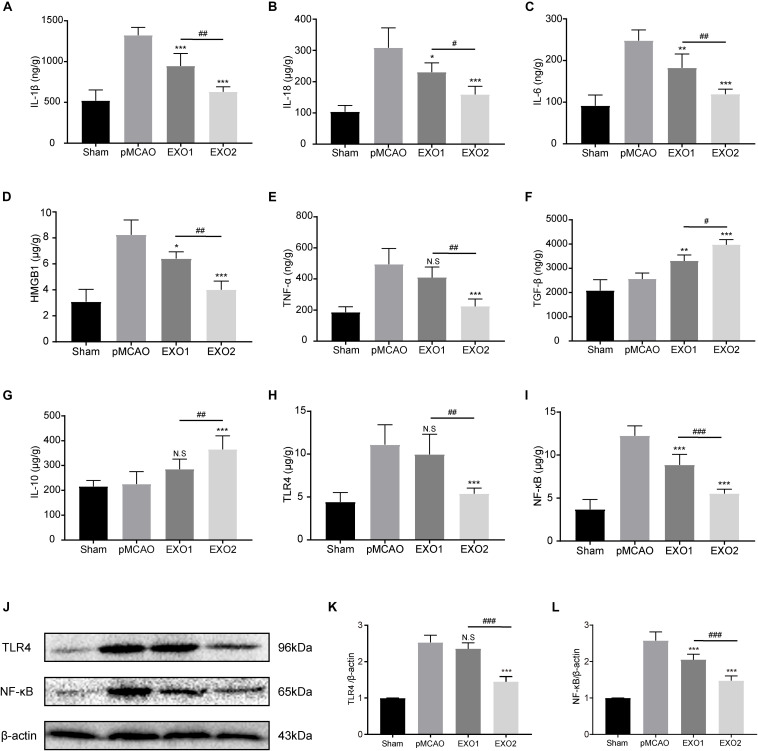
Basic plasma exosomes (EXO1) and melatonin-treated plasma exosomes (EXO2) reduced the expression of inflammatory cytokines in the cortex after permanent distal middle cerebral artery occlusion (pMCAO) through the TLR4/NF-kB pathway. **(A–G**) The concentrations of the inflammatory-associated cytokines in the cortex were detected through enzyme-linked immunosorbent assay (ELISA) at 72 h (*n* = 5). **(H–L)** The levels of TLR4 and NF-κB in the cortex were measured by ELISA and Western blotting at 72 h (*n* = 5). Data are presented as the mean ± SD. *P < 0.05, **P < 0.01, ***P < 0.001 vs. the pMCAO group. ^#^*P* < 0.05, ^##^*P* < 0.01, ^###^*P* < 0.001 vs. the EXO1 group.

Persistent neuroinflammation is commonly observed after ischemic stroke. To investigate the regulation of exosomes on the global inflammatory response after ischemic stroke, we examined the inflammatory and anti-inflammatory cytokine secretion profiles. Our data showed that basal plasma exosome administration effectively reduced the ischemia-induced inflammatory cytokine levels, including IL-1β, IL-18, IL-6, TNF-α, and HMGB1, and increased the anti-inflammatory cytokines TGF-β and IL-10 secretion, indicating its anti-inflammatory role in ischemic stroke (Figures 6A–G). MT-exosome treatment exhibited a more significant suppression of IL-1β, IL-18, IL-6, TNF-α, and HMGB1 secretions and increases in IL-10 and TGF-β, suggesting that melatonin enhances the anti-inflammatory potential of plasma exosomes.

The alternative expressions of the downstream inflammatory factors (IL-6, TNF-α, and HMGB1) indicated the possibility of regulating upstream regulatory elements. To investigate the regulatory mechanism of exosomes on the inflammatory response, the Toll-like receptors/nuclear factor kappa-B (TLR/NF-κB) pathway-associated proteins were detected. As shown in Figures 6H–L, the protein expressions of TLR4 and NF-κB p65 in the cerebral cortex increased significantly in the pMCAO group compared with those in the sham group (*P* < 0.01), while the administration of EXO1 greatly reduced the expression of NF-κB p65. In comparison with the EXO1 group, EXO2 treatment resulted in a more pronounced reduction in both TLR4 and NF-κB expressions (*P* < 0.05), suggesting that melatonin enhances the anti-inflammatory potential of plasma exosomes through the TLR4/NF-κB pathway.

### miRNA Expression Profiles in Adult Rat Plasma Exosomes

**FIGURE 7:**
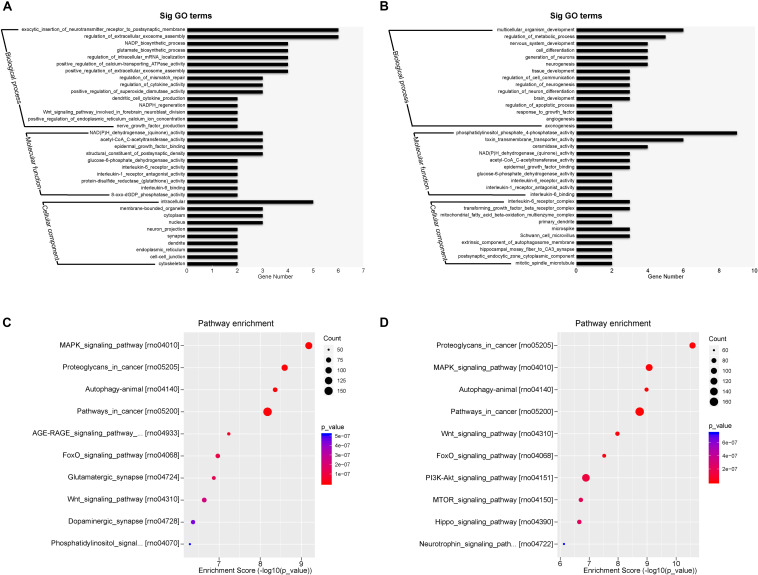
Gene Ontology (GO) classification and GO term enrichment of the top expressed microRNAs (miRNAs) in basal plasma exosomes and melatonin-treated exosomes. **(A,B)** Gene Ontology classification of the potential target genes cleaved by miRNA enrichment in basal plasma exosomes **(A)** and melatonin-treated exosomes **(B)** based on their involvement in various biological processes. **(C,D)** Pathway enrichment of the predicted targets of the differentially expressed miRNAs in basal plasma exosomes **(C)** and melatonin-treated exosomes **(D).**

The top expressed miRNAs from the plasma exosomes were evaluated and the GO functions of the target genes were analyzed. Figures 7A,B show the enrichment score values of the top significant enrichment terms (fold_enrichment) in the EXO1 and EXO2 groups. GO function analysis revealed that the miRNA target 1genes were involved in biological processes, cellular components, and molecular functions, indicating a comprehensive regulatory effect of miRNAs. Compared with the basic plasma exosome, the melatonin-treated exosomes exhibited more functions involved in neurological processes, including neurogenesis, neuron differentiation, apoptosis, and angiogenesis, which was in line with the neuroprotective effect of EXO2.

The most enriched miRNAs in EXO1 (Figure 7C) mainly participated in the MAPK signaling pathway, glutamatergic synapse, Wnt signaling pathway, dopaminergic synapse, and phosphatidylinositol signaling pathway. After melatonin treatment, the miRNA expression profiles were largely altered. Compared with the miRNAs in EXO1 (Figure 7D), the miRNA enrichment in EXO2 was more involved in the PI3K-Akt signaling pathway, MTOR signaling pathway, Hippo signaling pathway, and neurotrophin signaling pathway. These data suggest that melatonin treatment alters the miRNA composition and cellular functions, indicating its neural regulatory effects.

### Melatonin Alters MicroRNA Expression in Rat Plasma Exosomes

By detecting the miRNA variation profiles in the melatonin-treated plasma exosome, a total of 60 significantly differentially expressed miRNAs (*P*_FC_ ≤ 0.05) were identified (after removal of repetitive miRNAs). Detailed information regarding the 12 upregulated and 47 downregulated miRNAs is summarized in Tables 1, 2. Among these differentially expressed miRNAs, a certain number of miRNAs have been reported to be involved in neurological processes, including neural apoptosis, autophagy, inflammation, and angiogenesis.

**TABLE 1:** The upregulated miRNAs in the plasma exosome *via* melatonin treatment.

Mature_ID	log2FC	*P*-value	Involved neurological process	Resources
miR-129-2-3p	267.58	0.038	Autophagy	Sun et al., 2019
miR-496-3p	128.13	0.044		
miR-212-5p	23.27	0.029	Ferroptosis; neuroprotection	Xiao et al., 2019
miR-138-5p	15.97	0.041	Neuroprotection	Deng et al., 2019
miR-501-3p	8.17	0.023	AD serum biomarker	Hara et al., 2017
miR-363-3p	3.82	0.013	Cognitive function	Jiang et al., 2018
miR-451-5p	3.60	0.017		
miR-184	3.35	0.022	Neuroprotection; inflammation; anti-apoptosis	Chen and Stallings, 2007; McKiernan et al., 2012; Qin et al., 2015
miR-9a-5p	3.28	0.025	Anti-Inflammation; Neuroprotection	Yuze et al., 2020
miR-205	3.06	0.042	PD biomarker	Marques et al., 2017
miR-144-3p	2.87	0.046	Neuroprotection	Li et al., 2018
miR-92a-3p	2.85	0.040	Post-stroke depression	He et al., 2017

**TABLE 2:** The downregulated miRNAs in the plasma exosome via melatonin treatment.

Mature_ID	log2FC	*P*-value	Involved neurological process	Resources
miR-381-3p	–10.46	0.001		
miR-295-3p	–9.88	0.005		
miR-216b-5p	–9.76	0.005		
miR-217-5p	–9.62	0.0001		
miR-409a-5p	–9.37	0.007		
miR-216a-5p	–9.35	0.010	Microglia polarization	Liu et al., 2020
miR-770-3p	–8.82	0.015	Aging	Lee E.K. et al., 2018
miR-466b-3p	–8.80	0.021		
miR-466c-3p	–8.76	0.022		
miR-493-5p	–8.68	0.0002		
miR-193b-3p	–8.38	0.002	Ischemia marker	Chang and Lai, 2012
miR-483-5p	–8.38	0.007	Angiogenesis inhibitor	Qiao et al., 2011
miR-431	–8.37	0.031	Hypertension and vascular injury; neuroprotection	Han et al., 2018; Huo et al., 2019
miR-325-5p	–8.36	0.024		
miR-17-2-3p	–8.32	0.037	Oxidation; inflammation	Xu et al., 2018
miR-125a-3p	–8.19	0.027		
miR-342-5p	–8.11	0.028	Cell proliferation and differentiation; inflammation	Wei et al., 2013; Gao et al., 2017
miR-122-3p	–8.04	0.030		
miR-881-3p	–7.87	0.035		
miR-3065-5p	–7.79	0.042		
miR-204-3p	–7.67	0.038	AD plasma exosomal marker	Lugli et al., 2015
miR-136-5p	–7.60	0.001	Anti-inflammation	Zhong et al., 2019
miR-130b-5p	–7.57	0.012		
miR-485-5p	–7.39	0.049	Apoptosis; inflammation	Chen et al., 2016
miR-466b-2-3p	–7.12	0.019		
miR-466b-4-3p	–7.12	0.019		
miR-30b-3p	–7.12	0.026		
miR-1b	–6.99	0.0002		
miR-877	–6.49	0.020		
miR-299a-3p	–6.35	0.020		
miR-494-3p	–6.29	0.011	Neurotoxicity	Deng et al., 2020
miR-152-5p	–5.81	0.037		
miR-34b-5p	–5.45	0.041		
miR-206-3p	–5.42	0.011	Neuropathic pain	Wen et al., 2019
miR-497-5p	–3.69	0.043	BBB permeability	Zhu et al., 2019
miR-143-5p	–3.50	0.022		
miR-195-5p	–3.17	0.014	Anti-angiogenesis	Sandrim et al., 2016
miR-122-5p	–3.14	0.0003		
miR-134-5p	–2.88	0.048	Neuropathic pain	Zhu et al., 2019
miR-145-3p	–2.59	0.049	Macrophage polarization	Huang et al., 2019
miR-146b-5p	–2.50	0.003	Anti-inflammation	Chang and Lai, 2012
miR-199a-3p	–2.45	0.005		
miR-199a-5p	–2.42	0.008	Anti-inflammation; anti-apoptosis	Li M. et al., 2019; Yu et al., 2020
miR-143-3p	–2.33	0.006	Ischemia–reperfusion injury	Chang et al., 2019
miR-152-3p	–2.09	0.010	Anti-apoptosis; anti-oxidation	Zhang A. et al., 2019
miR-100-5p	–1.95	0.010		
miR-99a-5p	–1.64	0.028	Ischemic stroke biomarker	Zhao et al., 2017

*BBB, blood–brain barrier.*

**FIGURE 8:**
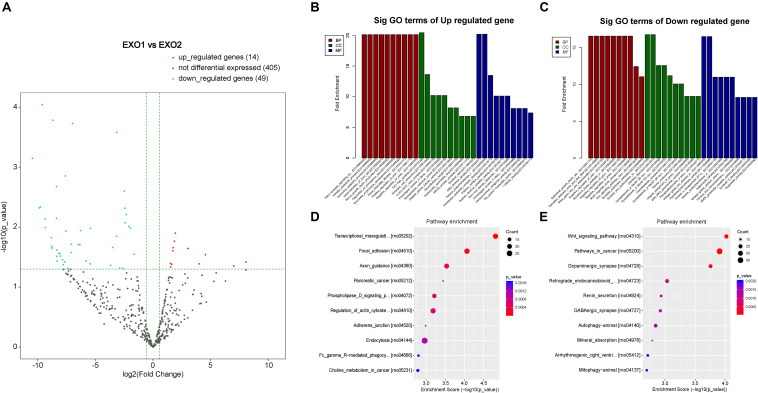
Volcano plot and Gene Ontology (GO) term enrichment of the differentially expressed microRNAs (miRNAs). **(A)** Volcano plot showing the melatonin-induced differential expression of the potential target miRNAs. **(B,C)** GO term enrichment for the predicted targets of the differentially expressed miRNAs. **(D,E)** Pathway enrichment of the predicted targets of the differentially expressed miRNAs.

To determine the involvement of these differentially expressed miRNAs in the neural recovery process, we performed a Gene Ontology classification based on their involvement in predicted cellular pathways. A volcano plot of the altered miRNAs is presented in Figure 8. GO classification of potential target genes and their involved biological processes are analyzed in Figures 8B–E. The GO classification revealed that altered miRNAs were involved in important processes such as nerve growth factor production, Wnt signaling pathway, and trans-synaptic signaling pathway (Figures 8B–E). A subsequent analysis of the modulation of these genes revealed the regulatory effect of melatonin through exosome secretion.

## Discussion

Inflammation plays an important role in secondary injury after permanent ischemic stroke. However, the detailed underlying mechanism of the inflammation process remains unclear. Recently, emerging studies have reported that pyroptosis, a caspase-1-dependent inflammasome-mediated programmed necrosis, greatly contributes to neuronal cell death and the inflammation response. Distinct from apoptosis and other types of programmed cell death, pyroptosis is a lytic process featuring cell swelling and large bubbles emanating from the plasma membrane. In cerebral ischemia, the NLR inflammasome assembles through the recognition of PAMPs and recruitment of pro-caspase-1 with the adapter protein ASC. Upon activation, active caspase-1 cleaves the precursor of IL-1β and IL-18 into their mature forms. In the meantime, cleavage of the N-terminal domain of GSDMD forms cellular membrane GSDMD pores, which subsequently release mature IL-1β and IL-18 into intercellular spaces and cause pyroptosis. In this study, we confirmed that intravenous injection of plasma exosomes effectively improved functional deficit recovery through the inhibition of neuronal cell pyroptosis and pyroptosis-induced inflammation. The results revealed that the administration of exosomes clearly inhibited caspase-1 activation, which subsequently prevented GSDMD cleavage and membrane GSMDM pore formation that leads to the secretion of inflammatory cytokines, including IL18, IL-1β, TNF-α, IL-6, and HMGB1.

Previous studies have shown that coalescing young and old vessels inhibited inflammation reactions in older mice (Wang et al., 2018; Zhang and Jin, 2020), indicating that there is an anti-inflammatory effect of plasma exosomes. In contrast to cell type-specific exosomes in a highly controlled process, the diversity of plasma cells determines its functional diversity. Growing evidence has shown that plasma exosomes deliver various proteins, lipids, nucleic acids, and small regulatory RNAs for cell communication and physiological regulation (Lee B.R. et al., 2018; Zhang and Jin, 2020). Zhou et al. reported that serum exosomes attenuate H_2_O_2_-induced apoptosis in rat H9C2 cells *via* ERK1/2 (Li P. et al., 2019). Riquelme et al. identified that endogenous plasma exosomes can communicate TLR4 relative signals and provide protection against ischemia and reperfusion injury in the myocardium (Minghua et al., 2018). However, to date, no studies have explored the biological function of plasma on cerebral ischemia. Here, we verified that plasma exosomes inhibited ischemia-induced inflammation through NF-κB induction. Meanwhile, melatonin treatment was found to enhance its therapeutic effect on ischemic stroke through the TLR4/NF-κB pathway. The internal molecular mechanism may lie in the specific composition of the exosomes.

To date, melatonin has demonstrated its diverse pharmacological functions against ischemic brain injury, including circadian rhythm regulation, anti-oxidation, anti-inflammation, and anti-apoptosis (Sinha et al., 2001; Feng et al., 2017). Nevertheless, whether melatonin stimulus acts in a paracrine fashion to exert its neuroprotective effect requires further investigation. It has been reported that melatonin reconditioning increases the expression of matrix metalloproteinase-9 (MMP-9) and MMP-13 and decreased the expression of TGF-β to restrain fibrosis (Saberi et al., 2019). In addition, melatonin-stimulated mesenchymal stem cell (MSC)-derived exosomes enhance functional recovery in acute liver ischemia–reperfusion injury, indicating that melatonin alters the exosome content for modulation of the microenvironment by paracrine mechanisms (Yoon et al., 2020). The present study revealed that melatonin enhanced the therapeutic effect of plasma exosomes on ischemia-induced inflammation and inflammation-dependent pyroptosis through the TLR4/NF-κB pathway, suggesting that component alteration and nerve beneficial substances are induced after melatonin administration. The downregulation of exosomal miR-100-5p and miR-199a-5p under melatonin treatment has been identified to directly regulate TLR4, suggesting the regulatory effects of melatonin-treated exosomal miRNAs. All of these results confirmed that melatonin might exert its neurological protection through the stimulation and alteration of the exosome content; nevertheless, due to the complicated composition of exosomes, we cannot exclude other mechanisms underlying its bioactivity in addition to miRNA differential expression.

As the main immune response cells in brain tissue, the status of the microglia determines the cerebral inflammation reaction. Studies have confirmed that ischemia stimulates microglial activation and promotes M1 polarization, in which the microglia secrete inflammatory mediators. With the occurrence of pyroptosis in the microglia, intracellular inflammatory mediators are largely released into the extracellular matrix, causing inflammatory injury and neuronal cell death in adjacent cells. Through an immunofluorescence assay, we confirmed the existence of pyroptosis in the microglia. The inflammatory cytokine secretion profiles demonstrated the anti-inflammatory and anti-pyroptosis activity of plasma exosomes, which eventually prevented adjacent neuronal cells from undergoing pyroptosis and improved nerve function recovery.

Exosomes manipulate intercellular communication through their embedded content, including proteins and regulatory non-coding RNAs. miRNAs have been considered as key and are the most studied regulatory molecules. The exosomal miRNAs that are involved in neuronal injury and inflammation signaling may provide key insights into the mechanisms involved in ischemic injury. With systematic investigation of the top-expressed miRNA profiles, we found that exosomal miRNAs are involved in various biological processes and molecular functions, including apoptosis, dopaminergic synapse, neurotrophin signaling pathway, Wnt signaling pathway, mTOR signaling pathway, and PI3K-Akt signaling pathway, suggesting regulatory roles in the neuroprotective effect against cerebral ischemia injury.

Based on studies indicating an altered miRNA profile upon external environmental stimulus, we extended plasma exosome therapy to tailor exosomal miRNA content. Identification of the differentially expressed miRNAs in the melatonin-treated plasma exosomes provided us with new directions for the exploration of therapeutic mechanisms. On analyzing the miRNA profiles, 12 upregulated and 47 downregulated miRNAs were discovered upon melatonin treatment. Among the upregulated miRNAs (Table 1), miR-212-5p was reported to attenuate ferroptotic cell death partially by targeting Ptgs2 in Neuro-2a cell lines (Xiao et al., 2019). The overexpression of miR-138-5p reduces neurological impairment and confers neuroprotection to astrocytes following ischemic stroke (Deng et al., 2019). miR-184 (Qin et al., 2015), miR-9a-5p (Yuze et al., 2020), and miR-144-3p (Li et al., 2018) have been reported to be involved in the inflammatory response and neuronal death against ischemic stroke. Some miRNAs, not present in EXO1, were upregulated in EXO2, suggesting that melatonin stimulates their transcription. Moreover, several miRNAs were downregulated in EXO2. Among the downregulated miRNAs, miR-17-3p was found to activate the Notch1/NF-κB pathways and downregulate the mitochondrial antioxidant enzymes Mn-SOD, Gpx2, and TrxR2 (Xu et al., 2018); miR-195-5p and miR-145-3p were reported to regulate macrophage polarization (Huang et al., 2019; Lin et al., 2019). Suppressing miR-152-3p protects against ischemia–reperfusion injuries *via* the activation of the AMPK/Foxo1 pathway (Zhang A. et al., 2019). These findings support and provide insights into the molecular mechanisms underlying enhancement in neurological recovery with melatonin treatment. However, further discussion of non-conformable cases is needed. miR-152-3p and miR-199a-5p, identified as neuronal protective molecules against cerebral ischemic injury or oxygen/glucose deprivation (OGD)-induced injury, were downregulated upon melatonin treatment (Li M. et al., 2019; Zhang A. et al., 2019), whereas miR-144-3p, upregulated with melatonin treatment, was shown to increase OGD/R-induced neuronal injury *via* Nrf-2/ARE signaling (Li et al., 2018). Similarly, the downregulation of miR-146b-5p after melatonin treatment was confirmed to protect oligodendrocyte precursor cells from oxygen/glucose deprivation-induced injury by regulating Keap1/Nrf2 signaling (Li X. et al., 2019). Despite their comprehensive biological functions, further mechanisms involved in the melatonin-enhanced neuroprotective effects by miRNA modulators and/or genetic regulators will be explored in our future work.

## Conclusion

In summary, our study confirmed that basal rat plasma exosomes attenuated the ischemic-induced inflammatory response, neuronal apoptosis, and inflammasome-dependent pyroptosis after stroke in rats and that melatonin significantly enhanced the therapeutic effect by regulating the TLR4/NF-κB pathway. Pretreatment with melatonin altered the microRNA profiles of the exosomes from rat plasma. The sequencing results identified effective miRNA components and their regulatory pathways involved in neurological recovery. However, the relationship between microRNAs and pyroptosis needs further research.

## Data Availability Statement

The sequencing data have been deposited in NCBI Gene Expression Omnibus (GEO, http://www.ncbi.nlm.nih.gov/geo/) with the accession number GSE147578.

## Ethics Statement

The animal study was reviewed and approved by the Ethics Committee of Wenzhou Medical University.

## Author Contributions

SY designed the experiment and wrote the manuscript. QZ guided and provided the funding. KW, JR, HZ, JC, HN, and XL performed the experiment and collected the data. LH, MW, and ZL analyzed the data and revised the manuscript. All authors contributed to the article and approved the submitted version.

## Conflict of Interest

The authors declare that the research was conducted in the absence of any commercial or financial relationships that could be construed as a potential conflict of interest.

## Footnotes

1 http://www.ncbi.nlm.nih.gov/geo/
